# Supplementary material for: Comparative genomics provides new insights into the diversity, physiology, and sexuality of the only industrially exploited tremellomycete: Phaffia rhodozyma
Source: BMC Genomics. 2016 Nov 9;17:901. doi: 10.1186/s12864-016-3244-7 (PMC5103461; doi:10.1186/s12864-016-3244-7)
Supplement: Additional file 6: — List of orphan genes with links to PFAM (related to Additional file 1: Table S1). (ZIP 1428 kb) [file 12864_2016_3244_MOESM6_ESM.zip › BLAST_HTML_FTR/G03566_P.html]

BLAST Search Results


```
BLASTP 2.2.27+


Reference:
Stephen F. Altschul, Thomas L. Madden, Alejandro A. Schäffer,
Jinghui Zhang, Zheng Zhang, Webb Miller, and David J. Lipman (1997),
"Gapped BLAST and PSI-BLAST: a new generation of protein database
search programs", Nucleic Acids Res. 25:3389-3402.


Reference for
composition-based statistics:
Alejandro A. Schäffer, L. Aravind, Thomas L. Madden, Sergei
Shavirin, John L. Spouge, Yuri I. Wolf, Eugene V. Koonin, and
Stephen F. Altschul (2001), "Improving the accuracy of PSI-BLAST
protein database searches with composition-based statistics and
other refinements", Nucleic Acids Res. 29:2994-3005.


Database: nr
           71,551,133 sequences; 26,053,659,533 total letters


Query= G03566_P

Length=92
                                                                      Score     E
Sequences producing significant alignments:                          (Bits)  Value

emb|CDZ98810.1|  hypothetical protein [Xanthophyllomyces dendrorh...   186    2e-58
ref|WP_013323092.1|  glycosyl transferase family 2 [Cyanothece sp...  37.0    1.7  
ref|XP_002174283.2|  hypothetical protein SJAG_03117 [Schizosacch...  36.2    2.5  
gb|KOM23918.1|  hypothetical protein XA68_1759 [Ophiocordyceps un...  35.0    6.2  


 >emb|CDZ98810.1| hypothetical protein [Xanthophyllomyces dendrorhous]
Length=91

 Score =  186 bits (471),  Expect = 2e-58, Method: Compositional matrix adjust.
 Identities = 91/91 (100%), Positives = 91/91 (100%), Gaps = 0/91 (0%)

Query  1   MSNGVKSALNSIKPSIKRQVFRPIDPTKLFKTQPPLYNNPIDINPNTHSSRWQIQEKQRR  60
           MSNGVKSALNSIKPSIKRQVFRPIDPTKLFKTQPPLYNNPIDINPNTHSSRWQIQEKQRR
Sbjct  1   MSNGVKSALNSIKPSIKRQVFRPIDPTKLFKTQPPLYNNPIDINPNTHSSRWQIQEKQRR  60

Query  61  VEKERQRLNRLDDMRDRVKQLEAQGWSMKKD  91
           VEKERQRLNRLDDMRDRVKQLEAQGWSMKKD
Sbjct  61  VEKERQRLNRLDDMRDRVKQLEAQGWSMKKD  91


>ref|WP_013323092.1| glycosyl transferase family 2 [Cyanothece sp. PCC 7822]
 gb|ADN14999.1| glycosyl transferase family 2 [Cyanothece sp. PCC 7822]
Length=880

 Score = 37.0 bits (84),  Expect = 1.7, Method: Composition-based stats.
 Identities = 26/79 (33%), Positives = 37/79 (47%), Gaps = 10/79 (13%)

Query  5    VKSALNSIKPSIKRQVFRPIDPTKLFKTQPPLYNNP-IDINPNTHSSRWQIQEKQR----  59
            ++  LN +   ++   F+ +DP  LF T   L NNP +D+NP  H  +W  +EKQ     
Sbjct  231  LEKTLNPLIHFLQIGSFQGLDPHSLFHTSHYLENNPGLDMNPLVHFIQWGAREKQDPFCY  290

Query  60   -----RVEKERQRLNRLDD  73
                   E E Q LN   D
Sbjct  291  AKLFPERENEDQSLNNSPD  309


>ref|XP_002174283.2| hypothetical protein SJAG_03117 [Schizosaccharomyces japonicus 
yFS275]
 gb|EEB07990.2| hypothetical protein SJAG_03117 [Schizosaccharomyces japonicus 
yFS275]
Length=256

 Score = 36.2 bits (82),  Expect = 2.5, Method: Compositional matrix adjust.
 Identities = 24/75 (32%), Positives = 35/75 (47%), Gaps = 9/75 (12%)

Query  11   SIKPSIKRQVFRPIDPTKLFKTQPPLYNNPIDINPNTHSSRWQIQEKQR---------RV  61
            ++KP++ R V +       F   PP    P+     T   R  IQ+KQR         R+
Sbjct  155  TVKPTVFRLVSKKTQENNGFHVVPPAGTKPVSSQKLTKKQRQNIQKKQREKAMKEEADRI  214

Query  62   EKERQRLNRLDDMRD  76
            ++ER R  RLD MR+
Sbjct  215  QRERLRSYRLDQMRE  229


>gb|KOM23918.1| hypothetical protein XA68_1759 [Ophiocordyceps unilateralis]
Length=327

 Score = 35.0 bits (79),  Expect = 6.2, Method: Composition-based stats.
 Identities = 26/81 (32%), Positives = 40/81 (49%), Gaps = 4/81 (5%)

Query  2    SNGVKSALNSIKPSIKRQVFR-PIDPTKLFKTQPPLYNNPIDINPNTHSSRWQIQEKQRR  60
            +NG K+  N   P  +  V + P DP   F    PL ++   +NP+   SR  +  + RR
Sbjct  73   TNGTKTMTNGSAPRRRPSVGKAPRDPRDRFA---PLGSSSPMVNPSDGLSRPGVGTRARR  129

Query  61   VEKERQRLNRLDDMRDRVKQL  81
             E + Q  +RL+ MR  V+ L
Sbjct  130  SEDDSQAQSRLERMRGAVRTL  150


Lambda      K        H        a         alpha
   0.321    0.135    0.405    0.792     4.96 

Gapped
Lambda      K        H        a         alpha    sigma
   0.267   0.0410    0.140     1.90     42.6     43.6 

Effective search space used: 648524678610


  Database: nr
    Posted date:  Sep 23, 2015 12:05 AM
  Number of letters in database: 26,053,659,533
  Number of sequences in database:  71,551,133


Matrix: BLOSUM62
Gap Penalties: Existence: 11, Extension: 1
Neighboring words threshold: 11
Window for multiple hits: 40
```
